# Supplementary material for: Hot spot prediction in protein-protein interactions by an ensemble system
Source: BMC Syst Biol. 2018 Dec 31;12(Suppl 9):132. doi: 10.1186/s12918-018-0665-8 (PMC6311905; doi:10.1186/s12918-018-0665-8)
Supplement: Supplementary file 2 — All relevant MATLAB code. A simple Matlab implement of our predictor. (ZIP 19,742 kb) [file 12918_2018_665_MOESM2_ESM.zip › Additional file 2/Read ASA/ASA website/dbMPIKT/5D3IB.html]

 NetSurfP - Protein Surface Accessibility and Secondary Structure Predictions

  

|  |  |  |
| --- | --- | --- |
|  |  | NetSurfP - Protein Surface Accessibility and Secondary Structure Predictions  Technical University of Denmark |

  


---

```
# For publication of results, please cite:
# A generic method for assignment of reliability scores applied to solvent accessibility predictions.
# Bent Petersen, Thomas Nordahl Petersen, Pernille Andersen, Morten Nielsen and Claus Lundegaard
# BMC Structural Biology 2009, 9:51 doi:10.1186/1472-6807-9-51
#
# Column 1: Class assignment - B for buried or E for Exposed - Threshold: 25% exposure, but not based on RSA
# Column 2: Amino acid
# Column 3: Sequence name
# Column 4: Amino acid number
# Column 5: Relative Surface Accessibility - RSA
# Column 6: Absolute Surface Accessibility
# Column 7: Z-fit score for RSA prediction
# Column 8: Probability for Alpha-Helix
# Column 9: Probability for Beta-strand
# Column 10: Probability for Coil
E G  5D3IB                  1    0.901  70.924  -1.226   0.003   0.003   0.994
E S  5D3IB                  2    0.680  79.673  -1.306   0.058   0.017   0.925
E M  5D3IB                  3    0.473  94.607  -1.372   0.115   0.016   0.868
E T  5D3IB                  4    0.454  62.998  -0.383   0.109   0.005   0.886
E P  5D3IB                  5    0.581  82.387  -0.577   0.406   0.004   0.590
E K  5D3IB                  6    0.477  98.119   0.875   0.502   0.002   0.495
B Y  5D3IB                  7    0.122  26.050   0.207   0.694   0.003   0.303
E E  5D3IB                  8    0.558  97.395   1.404   0.782   0.003   0.216
E D  5D3IB                  9    0.404  58.260   0.758   0.858   0.002   0.139
B L  5D3IB                 10    0.027   4.980   0.640   0.938   0.007   0.055
E R  5D3IB                 11    0.311  71.105   1.136   0.879   0.010   0.111
E A  5D3IB                 12    0.606  66.803   1.210   0.879   0.010   0.111
B Y  5D3IB                 13    0.176  37.547   1.058   0.802   0.014   0.185
B Y  5D3IB                 14    0.098  20.943   0.014   0.622   0.015   0.363
E T  5D3IB                 15    0.370  51.264  -0.689   0.268   0.043   0.689
E K  5D3IB                 16    0.508 104.454   0.007   0.053   0.043   0.903
E P  5D3IB                 17    0.551  78.159  -0.167   0.018   0.088   0.893
E S  5D3IB                 18    0.334  39.157  -1.469   0.020   0.205   0.775
B F  5D3IB                 19    0.181  36.246   0.026   0.022   0.552   0.426
E E  5D3IB                 20    0.393  68.640  -0.295   0.021   0.756   0.223
B F  5D3IB                 21    0.190  38.073   0.314   0.021   0.756   0.223
E E  5D3IB                 22    0.445  77.794  -0.049   0.002   0.816   0.182
E K  5D3IB                 23    0.336  69.136  -1.185   0.004   0.616   0.381
B Q  5D3IB                 24    0.279  49.901  -0.737   0.004   0.616   0.381
E F  5D3IB                 25    0.362  72.734  -0.896   0.004   0.616   0.381
B G  5D3IB                 26    0.216  17.023  -1.562   0.004   0.616   0.381
E F  5D3IB                 27    0.279  55.975  -0.223   0.002   0.816   0.182
B M  5D3IB                 28    0.201  40.240  -0.864   0.001   0.959   0.040
E L  5D3IB                 29    0.356  65.220  -0.304   0.001   0.900   0.099
E K  5D3IB                 30    0.332  68.272  -0.416   0.003   0.718   0.279
E P  5D3IB                 31    0.610  86.531  -0.903   0.004   0.197   0.799
E W  5D3IB                 32    0.591 142.232  -1.710   0.005   0.045   0.951
E T  5D3IB                 33    0.428  59.322  -1.941   0.005   0.045   0.951
E T  5D3IB                 34    0.397  55.119  -0.915   0.004   0.197   0.799
B V  5D3IB                 35    0.206  31.693  -0.820   0.004   0.514   0.481
B R  5D3IB                 36    0.195  44.632   0.854   0.003   0.718   0.279
B F  5D3IB                 37    0.101  20.371   0.448   0.002   0.816   0.182
B M  5D3IB                 38    0.040   7.984  -0.414   0.001   0.900   0.099
B N  5D3IB                 39    0.132  19.383   0.494   0.001   0.900   0.099
B V  5D3IB                 40    0.056   8.653   0.069   0.001   0.900   0.099
B I  5D3IB                 41    0.170  31.358   0.460   0.001   0.900   0.099
E P  5D3IB                 42    0.301  42.698  -0.222   0.004   0.420   0.576
E N  5D3IB                 43    0.489  71.531  -1.485   0.005   0.045   0.951
B R  5D3IB                 44    0.230  52.578  -0.802   0.005   0.262   0.733
B F  5D3IB                 45    0.229  46.041   0.028   0.003   0.718   0.279
B I  5D3IB                 46    0.073  13.542  -0.908   0.001   0.900   0.099
B Y  5D3IB                 47    0.065  13.784   0.283   0.011   0.918   0.071
B K  5D3IB                 48    0.128  26.412   0.802   0.011   0.918   0.071
B I  5D3IB                 49    0.044   8.177  -0.485   0.018   0.846   0.136
B A  5D3IB                 50    0.082   9.080  -1.128   0.023   0.655   0.322
B L  5D3IB                 51    0.099  18.072  -1.138   0.004   0.616   0.381
B V  5D3IB                 52    0.217  33.353   0.551   0.005   0.336   0.660
E G  5D3IB                 53    0.335  26.404  -0.433   0.052   0.084   0.864
E K  5D3IB                 54    0.675 138.745   0.114   0.113   0.043   0.844
E D  5D3IB                 55    0.461  66.459  -0.620   0.191   0.086   0.723
E E  5D3IB                 56    0.454  79.244  -0.435   0.191   0.086   0.723
E K  5D3IB                 57    0.532 109.391   0.229   0.278   0.093   0.628
E K  5D3IB                 58    0.507 104.331   1.388   0.278   0.093   0.628
B Y  5D3IB                 59    0.097  20.665  -0.216   0.191   0.086   0.723
E K  5D3IB                 60    0.464  95.465   0.112   0.191   0.086   0.723
E D  5D3IB                 61    0.597  86.057  -0.336   0.113   0.087   0.800
E G  5D3IB                 62    0.366  28.804  -0.519   0.052   0.084   0.864
E P  5D3IB                 63    0.548  77.761   0.381   0.052   0.084   0.864
E Y  5D3IB                 64    0.376  80.266  -0.553   0.019   0.141   0.840
E D  5D3IB                 65    0.408  58.750   0.159   0.018   0.088   0.893
E N  5D3IB                 66    0.290  42.456   0.459   0.005   0.336   0.660
B I  5D3IB                 67    0.018   3.386   1.157   0.003   0.718   0.279
B D  5D3IB                 68    0.065   9.294  -0.193   0.001   0.900   0.099
B V  5D3IB                 69    0.020   3.151   0.703   0.001   0.959   0.040
B F  5D3IB                 70    0.031   6.141   0.201   0.001   0.959   0.040
B I  5D3IB                 71    0.024   4.514   0.616   0.001   0.959   0.040
B V  5D3IB                 72    0.042   6.502   0.745   0.001   0.900   0.099
B L  5D3IB                 73    0.204  37.352   0.933   0.002   0.816   0.182
B E  5D3IB                 74    0.209  36.547  -0.164   0.004   0.514   0.481
E D  5D3IB                 75    0.464  66.805  -0.532   0.004   0.197   0.799
E N  5D3IB                 76    0.458  67.095  -1.691   0.004   0.197   0.799
E K  5D3IB                 77    0.648 133.211  -0.548   0.005   0.262   0.733
E Y  5D3IB                 78    0.387  82.702  -0.699   0.004   0.197   0.799
E Q  5D3IB                 79    0.592 105.821  -0.576   0.005   0.336   0.660
B L  5D3IB                 80    0.212  38.836  -1.089   0.003   0.718   0.279
B K  5D3IB                 81    0.166  34.084  -0.404   0.001   0.900   0.099
E K  5D3IB                 82    0.291  59.838   0.430   0.001   0.959   0.040
B Y  5D3IB                 83    0.096  20.601   0.723   0.001   0.959   0.040
B S  5D3IB                 84    0.122  14.322  -0.493   0.001   0.959   0.040
B V  5D3IB                 85    0.174  26.698  -0.841   0.003   0.718   0.279
B G  5D3IB                 86    0.123   9.641  -2.464   0.019   0.141   0.840
B G  5D3IB                 87    0.219  17.259  -1.785   0.022   0.359   0.619
B I  5D3IB                 88    0.084  15.558  -0.143   0.002   0.816   0.182
B T  5D3IB                 89    0.180  24.924   0.973   0.002   0.816   0.182
E K  5D3IB                 90    0.340  69.917   0.457   0.023   0.655   0.322
E T  5D3IB                 91    0.384  53.261  -0.051   0.022   0.359   0.619
E N  5D3IB                 92    0.272  39.835   0.089   0.020   0.205   0.775
E S  5D3IB                 93    0.650  76.121  -0.860   0.020   0.205   0.775
E K  5D3IB                 94    0.618 127.205  -0.406   0.019   0.141   0.840
E K  5D3IB                 95    0.673 138.498  -0.608   0.018   0.088   0.893
E V  5D3IB                 96    0.341  52.427  -0.253   0.018   0.047   0.935
E N  5D3IB                 97    0.502  73.464  -1.651   0.018   0.088   0.893
E H  5D3IB                 98    0.543  98.826  -0.180   0.020   0.205   0.775
E K  5D3IB                 99    0.271  55.806  -0.343   0.004   0.420   0.576
B V  5D3IB                100    0.087  13.372   0.134   0.004   0.616   0.381
B E  5D3IB                101    0.252  43.972  -0.897   0.002   0.816   0.182
B L  5D3IB                102    0.032   5.896   0.179   0.002   0.816   0.182
B S  5D3IB                103    0.138  16.150  -0.246   0.001   0.900   0.099
B I  5D3IB                104    0.027   5.013  -0.201   0.001   0.900   0.099
B T  5D3IB                105    0.229  31.762  -0.066   0.001   0.900   0.099
B K  5D3IB                106    0.193  39.638   0.095   0.001   0.900   0.099
E K  5D3IB                107    0.427  87.813  -0.401   0.003   0.718   0.279
E D  5D3IB                108    0.554  79.831  -0.565   0.021   0.279   0.699
E N  5D3IB                109    0.623  91.236  -1.043   0.018   0.088   0.893
E Q  5D3IB                110    0.596 106.481   0.327   0.020   0.205   0.775
E G  5D3IB                111    0.601  47.338  -0.158   0.005   0.336   0.660
E M  5D3IB                112    0.448  89.565   0.161   0.004   0.514   0.481
E I  5D3IB                113    0.378  69.856  -0.326   0.004   0.616   0.381
E S  5D3IB                114    0.327  38.313  -0.785   0.004   0.514   0.481
E R  5D3IB                115    0.401  91.829  -0.742   0.004   0.197   0.799
E D  5D3IB                116    0.477  68.736  -1.100   0.004   0.085   0.910
E V  5D3IB                117    0.441  67.751  -0.816   0.020   0.205   0.775
E S  5D3IB                118    0.463  54.299  -1.090   0.021   0.279   0.699
E E  5D3IB                119    0.362  63.206   0.137   0.004   0.616   0.381
B Y  5D3IB                120    0.079  16.968  -0.255   0.002   0.816   0.182
E M  5D3IB                121    0.375  75.078   0.492   0.001   0.900   0.099
B I  5D3IB                122    0.072  13.357  -1.854   0.002   0.816   0.182
E T  5D3IB                123    0.580  80.460  -0.097   0.004   0.420   0.576
E K  5D3IB                124    0.417  85.859   0.040   0.020   0.205   0.775
E E  5D3IB                125    0.533  93.028  -0.049   0.022   0.359   0.619
E E  5D3IB                126    0.468  81.812   0.569   0.023   0.655   0.322
B I  5D3IB                127    0.064  11.766   0.330   0.085   0.775   0.140
B S  5D3IB                128    0.176  20.580   0.509   0.087   0.683   0.230
B L  5D3IB                129    0.085  15.490  -0.413   0.252   0.423   0.325
E K  5D3IB                130    0.334  68.745   0.570   0.352   0.332   0.316
E E  5D3IB                131    0.378  66.037   0.198   0.538   0.173   0.289
B L  5D3IB                132    0.077  14.154   0.621   0.660   0.049   0.291
B D  5D3IB                133    0.099  14.309  -0.329   0.831   0.044   0.125
B F  5D3IB                134    0.183  36.668   0.255   0.938   0.007   0.055
E K  5D3IB                135    0.352  72.509   1.549   0.975   0.003   0.022
B L  5D3IB                136    0.040   7.397   0.643   0.975   0.003   0.022
B R  5D3IB                137    0.158  36.113   0.963   0.975   0.003   0.022
E K  5D3IB                138    0.409  84.090   1.078   0.975   0.003   0.022
E Q  5D3IB                139    0.292  52.080   1.451   0.975   0.003   0.022
B L  5D3IB                140    0.041   7.580   0.415   0.938   0.007   0.055
B I  5D3IB                141    0.168  31.099   0.521   0.938   0.007   0.055
E E  5D3IB                142    0.640 111.808   0.689   0.879   0.010   0.111
E K  5D3IB                143    0.483  99.415   0.449   0.717   0.014   0.269
B H  5D3IB                144    0.248  45.184   0.838   0.561   0.047   0.393
E N  5D3IB                145    0.489  71.531   0.179   0.386   0.097   0.517
B L  5D3IB                146    0.113  20.617  -0.114   0.278   0.093   0.628
B Y  5D3IB                147    0.207  44.129   0.075   0.199   0.152   0.649
E G  5D3IB                148    0.554  43.568  -0.354   0.118   0.150   0.732
E N  5D3IB                149    0.528  77.328  -1.352   0.056   0.142   0.802
B M  5D3IB                150    0.290  58.049  -0.443   0.018   0.088   0.893
E G  5D3IB                151    0.600  47.181  -1.270   0.018   0.088   0.893
E S  5D3IB                152    0.498  58.366   0.025   0.005   0.045   0.951
B G  5D3IB                153    0.183  14.394  -1.976   0.005   0.262   0.733
E T  5D3IB                154    0.354  49.141   1.343   0.002   0.816   0.182
B I  5D3IB                155    0.049   9.009   0.638   0.001   0.959   0.040
E V  5D3IB                156    0.271  41.730   1.542   0.001   0.959   0.040
B I  5D3IB                157    0.034   6.346   0.127   0.001   0.959   0.040
B K  5D3IB                158    0.210  43.197   0.551   0.001   0.959   0.040
B M  5D3IB                159    0.101  20.250  -0.206   0.002   0.816   0.182
E K  5D3IB                160    0.516 106.162   0.105   0.005   0.262   0.733
E N  5D3IB                161    0.663  97.078  -1.666   0.005   0.045   0.951
E G  5D3IB                162    0.631  49.628  -2.133   0.005   0.015   0.979
E G  5D3IB                163    0.527  41.491  -0.421   0.004   0.085   0.910
E K  5D3IB                164    0.496 101.965   0.865   0.004   0.616   0.381
B Y  5D3IB                165    0.202  43.082   1.001   0.002   0.816   0.182
E T  5D3IB                166    0.400  55.522   0.101   0.011   0.918   0.071
B F  5D3IB                167    0.120  24.084   0.776   0.018   0.846   0.136
E E  5D3IB                168    0.355  61.949  -0.455   0.021   0.756   0.223
B L  5D3IB                169    0.094  17.156   0.095   0.064   0.216   0.721
E H  5D3IB                170    0.337  61.373  -0.383   0.113   0.087   0.800
E K  5D3IB                171    0.580 119.265   0.530   0.118   0.150   0.732
E K  5D3IB                172    0.334  68.704   0.520   0.199   0.152   0.649
B L  5D3IB                173    0.084  15.307   0.575   0.118   0.150   0.732
E Q  5D3IB                174    0.476  84.924   0.574   0.056   0.142   0.802
E E  5D3IB                175    0.477  83.262   0.130   0.118   0.150   0.732
E H  5D3IB                176    0.394  71.632  -0.637   0.113   0.087   0.800
E R  5D3IB                177    0.524 119.904  -0.452   0.113   0.043   0.844
E M  5D3IB                178    0.482  96.548  -1.119   0.053   0.043   0.903
E A  5D3IB                179    0.490  53.965  -0.989   0.053   0.043   0.903
E D  5D3IB                180    0.498  71.819  -0.095   0.019   0.141   0.840
E V  5D3IB                181    0.333  51.182   0.311   0.022   0.359   0.619
B I  5D3IB                182    0.085  15.688  -0.175   0.021   0.451   0.528
E D  5D3IB                183    0.524  75.508   0.559   0.021   0.279   0.699
B G  5D3IB                184    0.176  13.875  -0.551   0.018   0.088   0.893
E T  5D3IB                185    0.692  95.939   0.211   0.018   0.088   0.893
E N  5D3IB                186    0.559  81.808   0.762   0.019   0.141   0.840
B I  5D3IB                187    0.100  18.537   0.214   0.005   0.336   0.660
E D  5D3IB                188    0.481  69.283   0.529   0.004   0.420   0.576
E N  5D3IB                189    0.433  63.420   0.957   0.004   0.616   0.381
B I  5D3IB                190    0.065  11.969   1.066   0.002   0.816   0.182
B E  5D3IB                191    0.252  44.042  -0.042   0.001   0.900   0.099
B V  5D3IB                192    0.077  11.773  -0.351   0.001   0.900   0.099
E N  5D3IB                193    0.360  52.645   0.758   0.001   0.900   0.099
B I  5D3IB                194    0.117  21.608   0.132   0.003   0.718   0.279
E K  5D3IB                195    0.745 153.329   1.154   0.005   0.015   0.979

Explain the output.  Go back.
```

---
